# Obstructive Sleep Apnea and Subclinical Ischemic Heart Disease Risk in Obese Adults: Findings from an International Cross-Sectional Study

**DOI:** 10.14789/ejmj.JMJ25-0041-OA

**Published:** 2026-01-21

**Authors:** CHINENYE IGUH, EDITH NWANDIKO, ASYA AZAD, BEESHAM KUMAR, MARVELOUS OLUWADAMILARE POPOOLA, GEOFFERY IGBERAESE, QUDSIAH GHAZANFAR, OLUWADAMILOLA ONANUGA, RACHA AL NIAZI, MEERA AL SHAMSI, KAINAT HABIB

**Affiliations:** 1 Department of Psychiatry and Neurology, Windsor University School of Medicine, Basseterre, St. Kitts and Nevis (West Indies); 1 Department of Psychiatry and Neurology, Windsor University School of Medicine, Basseterre, St. Kitts and Nevis (West Indies); 2 Department of Medicine, Wrexham Maelor Hospital, Wrexham, United Kingdom; 2 Department of Medicine, Wrexham Maelor Hospital, Wrexham, United Kingdom; 3 Department of Medicine, Tameside General Hospital, Manchester, United Kingdom; 3 Department of Medicine, Tameside General Hospital, Manchester, United Kingdom; 4 Department of Medicine, Jinnah Medical and Dental College, Karachi, Pakistan; 4 Department of Medicine, Jinnah Medical and Dental College, Karachi, Pakistan; 5 Department of Accident and Emergency, Calvary Adelaide Hospital, Adelaide, Australia; 5 Department of Accident and Emergency, Calvary Adelaide Hospital, Adelaide, Australia; 6 Department of Psychiatry, Middleton St. George Priory, Middleton St. George, United Kingdom; 6 Department of Psychiatry, Middleton St. George Priory, Middleton St. George, United Kingdom; 7 Department of Medicine, Rawalpindi Medical University, Rawalpindi, Pakistan; 7 Department of Medicine, Rawalpindi Medical University, Rawalpindi, Pakistan; 8 Department of Medicine, Nottingham University Hospitals NHS Trust, Nottingham, United Kingdom; 8 Department of Medicine, Nottingham University Hospitals NHS Trust, Nottingham, United Kingdom; 9 Department of Orthodontics, Dubai Health Authority, United Arab Emirates; 9 Department of Orthodontics, Dubai Health Authority, United Arab Emirates; 10 Department of Internal Medicine, Zayed Higher Organization for People of Determination, Abu Dhabi, United Arab Emirates; 10 Department of Internal Medicine, Zayed Higher Organization for People of Determination, Abu Dhabi, United Arab Emirates

**Keywords:** obesity, obstructive sleep apnea, ischemic heart disease, STOP-Bang questionnaire, INTERHEART Risk Score

## Abstract

**Objectives:**

The purpose of this study was to determine the relationship between OSA and subclinical risks of IHD in obese adults.

**Materials and Methods:**

A cross-sectional survey was conducted among 500 obese adults attending outpatient clinics in international medical centers. A structured questionnaire, comprising the STOP-Bang Questionnaire (SBQ) for OSA risk assessment and the INTERHEART Modifiable Risk Score (IHMRS) for subclinical IHD risk, was used to collect data. SPSS v.26 was used to perform statistical analyses.

**Results:**

The majority of participants were men (67%), aged between 50 and 59 years (53%). The OSA was moderately positively correlated with the risk of subclinical IHD (r = 0.36, p < 0.001). The male scores were higher in both STOP-Bang and IHMRS (p < 0.01) and were associated with older age (p < 0.001). Regression analysis revealed that OSA risk (B = 0.32, p = 0.001), older age (B = 0.41, p = 0.002), male gender, diabetes, blood pressure, and family history of heart disease were significant predictors of subclinical IHD risk. In contrast, physical activity was protective (B = -0.42, p = 0.009).

**Conclusions:**

Obese adults had a significant association between OSA risk and subclinical IHD risk. The most important predictors were OSA, age, male sex, diabetes, hypertension, and family history, with physical activity exhibiting a protective effect. The burden of overt cardiovascular disease can be alleviated by early detection and combined treatment of OSA and cardiovascular risks in obese persons.

## Introduction

Obesity is a major epidemic of the 21st century, contributing to dyslipidemia, hypertension, and type 2 diabetes. It affects nearly one-quarter of adults worldwide and is strongly linked to cardiometabolic diseases. Excess visceral fat is central to their development^1, 2)^. Obstructive sleep apnea (OSA) and obesity are closely related, sharing mechanisms that drive cardiometabolic and psychological disorders^3)^.

OSA is prevalent in obesity and, in a series of mechanisms including intermittent hypoxia and sleep fragmentation, exacerbates dysfunction in adipose tissue and cardiometabolic risk^4)^. Obstructive sleep apnea (OSA) is a sleep-related breathing disorder characterized by the periodic blockage of the upper airway during sleep, resulting in intermittent hypoxia and disrupted sleep^5)^. Notably, OSA has only one reversible risk factor, obesity, and both conditions share pathophysiological pathways and comorbidities, particularly cardiovascular diseases, which highlights the importance of integrating management approaches^6)^.

Obesity raises the risk of ischemic heart disease (IHD) regardless of whether one is metabolically healthy or not, which disputes the idea of metabolically healthy obesity^7)^. Ischemic heart disease (IHD) is a complication of atherosclerosis or functional coronary changes and is currently categorised as acute or chronic coronary syndrome^8)^.

Cardiovascular disease (CVD) risk factors can be changed, including physical inactivity, poor diet, tobacco use, hypertension, and obesity, whereas non-modifiable risk factors are age, gender, family history, and ethnicity. It should be emphasised that obesity and overweight alone are risk factors leading to myocardial infarction and ischemic heart disease, without metabolic syndrome^9, 10)^. In addition, it has been shown that high BMI and low aerobic fitness during adolescence alone forecast IHD in adulthood, highlighting the long-term effects of early-life exposures^11)^.

Due to the overlap of risk factors and mechanisms, the clinical importance of studying the relationship between OSA and the subclinical risk of IHD in obese adults is significant. This relationship can be used to identify high-risk individuals early, improve preventive cardiology care, and advocate a multidisciplinary approach to obesity. Even though obesity is dominant and OSA is underdiagnosed, the connection between OSA and subclinical IHD is not an adequately investigated topic. Hypertension, inflammation, and metabolic imbalance are generally attributed to both conditions. The study was focused on evaluating the interrelation between OSA and subclinical IHD risk in obese adults in global health care facilities. It also analysed demographic and clinical characteristics, including age, gender, and comorbidity, that are linked to elevated OSA risk.

## Materials and Methods

The current study used a cross-sectional study to identify the association between the risk of obstructive sleep apnea (OSA) and the subclinical risk of ischemic heart disease (IHD) across international medical centers. Outpatients from selected hospitals in various countries were recruited as participants. Since the authors were affiliated with different countries, data were collected from a wide range of locations, enhancing the diversity and representativeness of the sample. Data regarding demographic and clinical characteristics, OSA risk, and subclinical IHD risk were gathered using a structured questionnaire. The trained research assistants approached the participants during their usual clinic visits, explained the purpose of the study, allowed them time to discuss any questions or concerns, and then obtained consent. This methodology ensured that the process was straightforward, culturally sensitive, and respectful of the participants, without compromising the quality of data collection.

### Recruitment and sample

This study was based on an infinite population, where the estimated prevalence (p) was 0.5 in the absence of local data, assuming a 95% confidence interval (Z = 1.96) and a margin of error = 0.05^12)^. The convenience sampling technique was used to recruit obese adults from the outpatient clinics of selected hospitals across multiple countries. A total of 550 people were approached, and 50 of them were ineligible or refused to participate. The remaining 500 participants provided informed consent, underwent the study assessment, and were included in the final analysis.

### Eligibility criteria

The study included adults aged 18 years and above with a body mass index (BMI) of 30 kg/m^2^ or higher, who were considered obese, willing to provide informed consent, and attending outpatient clinics. Patients with a previously diagnosed CVD history (myocardial infarction, angina, or heart failure) were excluded in favor of subclinical ischemic heart disease. Patients under active therapy for obstructive sleep apnea, pregnant women, and those with a severe systemic illness or condition potentially impacting study inclusion, such as advanced renal or hepatic disease, were excluded, as well. Moreover, participants who were unable to complete the study assessments due to cognitive or communication impairments were excluded from the study. The criteria were established to ensure that the obese adult study population accurately represented the risk of OSA and subclinical IHD, and to control for confounding factors.

### Assessment tools and Procedure

Data for this study were collected using a structured questionnaire, which consisted of three main sections: demographic data, the STOP-Bang Questionnaire (SBQ), and the INTERHEART Modifiable Risk Score (IHMRS). The demographic section comprised items such as age, gender, marital status, educational level, residence, smoking status, diabetes, hypertension, family history of heart disease, and weekly physical activity. It also included other relevant medical and lifestyle characteristics of the respondents.

The SBQ assessed the risk of OSA. It is a validated screening tool created by Chung et al. in 2008 and is widely used in clinical practice. The questionnaire measures eight items: snoring, tiredness, observed apnea, high blood pressure, body mass index, age, neck circumference, and gender. These items are rated as 0 (absent) or 1 (present). The total score ranges from 0 to 8. The participants are classified into three categories based on their risk of OSA: low risk (0-2), intermediate risk (3-4), and high risk (5-8). The tool exhibits high internal consistency, with a Cronbach's alpha value of 0.70 to 0.75. It is concise and user-friendly, with high predictive validity, making it effective in clinical settings. The original authors granted permission to use the SBQ, and the original English version was used without cultural and linguistic translation in this research^13)^. The current study also utilized the IHMRS, an instrument developed by Yusuf et al. (2004) for the original INTERHEART study and subsequently validated in 2010. The IHMRS classifies the risk of a person having cardiovascular issues in relation to modifiable lifestyle- related conditions, such as smoking, hypertension, diabetes, body weight, diet, physical activity, stress, and alcohol consumption. The IHMRS is viewed as a valuable and effective measure of cardiovascular risk because it is easy to use and reproducible. The use permission of the IHMRS was obtained before application, and the original English version was used in the work without cultural and linguistic adaptation^14)^.

Research assistants were trained, the study's objectives were explained, and informed consent was obtained in writing. The data was collected between February 2025 and August 2025. Upon consent, participants were given a structured questionnaire that encompassed demographic data, the SBQ to assess the risk of OSA, and the INTERHEART Modifiable Risk Score (IHMRS) to determine the risk of subclinical ischemic heart disease based on modifiable lifestyle behaviors. The questionnaire was filled in by the participants themselves or with the help of the research assistant, who clarified the questions when necessary (depending on literacy level). The research assistants maintained cultural sensitivity and adhered to ethical considerations. All completed questionnaires were verified as complete and stored for analysis.

### Statistical plan

Data were analysed using IBM SPSS Statistics version 26 (IBM Corp.). Participants' demographics were summarised using descriptive statistics, presented in frequencies and percentages. Kolmogorov-Smirnov and Shapiro-Wilk tests were used to test the normality of the main study variables. Pearson correlation coefficients were used to assess associations between the SBQ and the INTERHEART Risk Score (IHRS). Independent samples t-tests were used to analyse differences in gender in the context of SBQ and IHRS scores. In contrast, one-way ANOVA was used to compare differences between age groups. The predictors of IHRS scores, including SBQ, demographic, and clinical risk factors, were identified using multiple linear regression. The relationship between a family history of heart disease and levels of physical activity was also tested using the chi-square test. All statistical tests were two-tailed, with significance set at p < 0.05.

### Research ethics

The study was conducted in accordance with the ethical standards of research involving human participants. The data collection process was initiated following ethical approval by the Institutional Review Board (032/RMU/IRB/2025) of the Rawalpindi Medical University. This approval ensured that the study adhered to the principles of respect for participants, beneficence, and confidentiality. Before the commencement of the study, all participants were informed of the study's objectives, procedures, risks, and benefits. Participation was voluntary, and written informed consent was obtained before participation. The participants were told that they could withdraw from the study at any time without any adverse effects. All personal information was processed safely and used confidentially for research purposes. Missing or incomplete data were handled cautiously, and non-essential missing values were managed through pairwise deletion throughout the analysis process. Participants with less than 20% survey responses were eliminated to ensure the validity and reliability of the data. All these steps were taken to maintain the integrity of the research and safeguard the rights and privacy of the participants.

## Results

### Participant characteristics

The demographics of 500 participants are given in Table 1. The majority of the participants were 50-59 years old (267, 53%), followed by those 60 years or older (111, 22%), and then 18-39 years old (25, 5%). There were 334 males (67%) and 166 females (33%). About marital status, 229 (46%) were married, 108 (22%) were single, and 163 (32%) were divorced or separated. The highest educational attainment was higher secondary/intermediate education (175, 35%), and 64 (13%) had no formal education. The majority of the participants (311, 62%) were residing in rural regions. About the lifestyle, 230 (46%) had formerly smoked, and 205 (41%) were current smokers, with 65 (13%) having never smoked. There were high levels of chronic conditions: diabetes (348, 70%), hypertension (368, 74%), and family history of heart disease (333, 67%). Physical activity was light (280, 56%), and a smaller number of participants were sedentary (145, 29%), moderately active (61, 12%), or vigorously active (14, 3%).

**Table 1:** Baseline demographic and clinical characteristics of participants (N = 500)

Variable	f (N)	%		Variable	f (N)	%
Age				Smoking status		
18-29 years	5	1		Never smoked	65	13
30-39 years	20	4		Former smoker	230	46
40-49 years	97	19		Current smoker	205	41
50-59 years	267	53		Diabetes		
60 years or above	111	22		Yes	348	70
Gender				No	152	30
Male	334	67		Hypertension		
Female	166	33		Yes	368	74
Marital status				No	132	26
Single	108	22		Family history of heart disease		
Married	229	46		Yes	333	67
Divorced/Separated	163	32		No	167	33
Educational level				Weekly physical activity		
No formal education	64	13		Sedentary (little/no exercise)	145	29
Primary	73	15		Light (< 150 min/week)	280	56
Secondary	121	24		Moderate (≥ 150 min/week)	61	12
Higher secondary/Intermediate	175	35		Vigorous	14	3
Graduate or above	67	13				
Residence						
Urban	189	38				
Rural	311	62				

Note. f = frequency, % = percentage; Values are presented as N (%), N = 500; No statistical comparisons were performed for demographic variables in this table

### Normality testing of study variables

Table 2 presents the findings of the normality tests on the study variables. The p-values in Kolmogorov- Smirnov (SBQ: 0.200; IHRS: 0.200) and Shapiro- Wilk tests (SBQ: 0.731; IHRS: 0.648) were greater than 0.05, meaning that the data were normally distributed in both the Stop Bang Questionnaire (SBQ) and the INTERHEART Risk Score Questionnaire (IHRS).

**Table 2:** Normality tests for STOP-Bang and INTERHEART Risk Scores (N = 500)

Variable	Kolmogorov-Smirnov statistic	df	p	Shapiro- Wilk statistic	df	p
Stop bang questionnaire (SBQ)	0.021	500	0.200	0.998	500	0.731
INTERHEART Risk Score questionnaire (IHRS)	0.024	500	0.200	0.997	500	0.648

Note. Kolmogorov-Smirnov test used Lilliefors significance correction; df = degree of freedom; A p-value > 0.05 was considered statistically significant, indicating normal distribution; N = 500.

### Correlation between OSA risk and subclinical IHD risk

A moderate positive correlation is observed between the SBQ and the INTERHEART Risk Score (IHRS), with a correlation coefficient of r = 0.36 (p < 0.001), as shown in Table 3. This indicates that a greater OSA risk measured by SBQ is linked to a greater risk of subclinical ischemic heart disease according to the IHRS.

**Table 3:** Correlation between STOP-Bang and INTERHEART Risk Scores among participants (N = 500)

Variables	1	2
Stop bang questionnaire (SBQ)	-	0.36**
INTERHEART Risk Score questionnaire (IHRS)	-	-

Note. Values represent Pearson correlation coefficients (r) between continuous variables; N = 500; p < 0.001 (2-tailed) was considered statistically significant and is denoted with double asterisks (**).

### Gender differences in OSA and subclinical IHD risk

Table 4 indicates a significant gender variation in the STOP-BANG (SBQ) and INTERHEART Risk Score (IHRS). The males had higher SBQ scores (12.38 ± 1.45) compared to females (11.74 ± 1.75), t = -4.05, p = 0.001, with a small-to-moderate effect size (Cohen's d = 0.39), indicating a higher risk of OSA among the males. In a similar measure, males (35.89 ± 2.86) had a higher IHRS score compared to females (35.08 ± 3.11), t = 2.89, p = 0.004, with a small effect size (Cohen d = 0.27) indicating a greater risk of subclinical ischemic heart disease in males.

**Table 4:** Gender differences in STOP-Bang and INTERHEART Risk Scores (N = 500)

Variable	Male (N = 334; 67%) M ± S.D	Female (N = 166; 33%) M ± S.D	t	p	Cl 95% LL	UL	Cohen's D
Stop bang questionnaire (SBQ)	12.38 ± 1.45	11.74 ± 1.75	-4.05	< 0.001**	-0.95	-0.33	0.39
INTERHEART Risk Score questionnaire (IHRS)	35.89 ± 2.86	35.08 ± 3.11	2.89	0.004**	0.26	1.36	0.27

Note. Values are presented as Mean ± Standard Deviation; Independent samples t-tests were conducted to compare participants of both genders; Group sizes are shown as N (%); Reported statistics include p-values, t-values, 95% Confidence Intervals (CI), and effect sizes (Cohen’s d); A p-value < 0.01 was considered statistically significant, N = 500.

### Age-related differences in OSA and subclinical IHD risk

Table 5 presents the significant differences in STOP-BANG (SBQ) and INTERHEART Risk Score (IHRS) between the age groups. SBQ scores rose as age increased, with 10.2 ± 0.8 in individuals aged 18-29 years, 12.7 ± 1.5 in the 50-59 years group, and then a slight decline in those aged 60-65 years and above (12.4 ± 1.4), with F (4, 495) = 9.75, p = 0.001, and η^2^ = 0.073. Likewise, there was a steady increase in the IHRS scores between the youngest group with 33.5 ± 2.4 and the remaining groups with 37.1 ± 2.7, F (4,495) = 8.35, p = 0.001, and η^2^ = 0.063. The results revealed that OSA risk and the subclinical ischemic heart disease risk both increase with age.

**Table 5:** Age group differences in STOP-Bang and INTERHEART Risk Scores (N = 500)

Variable	18-29 years (N = 5; 1%) M ± S.D	30-39 years (N = 20; 4%) M ± S.D	40-49 years (N = 97; 19%) M ± S.D	50-59 years (N = 267; 53%) M ± S.D	60 years & above (N = 111; 22%) M ± S.D	F(4,495)	p	η^2^
Stop bang questionnaire (SBQ)	10.2 ± 0.8	11.2 ± 1.4	12.5 ± 1.6	12.7 ± 1.5	12.4 ± 1.4	9.75	< 0.001**	0.073
INTERHEART Risk Score questionnaire (IHRS)	33.5 ± 2.4	34.8 ± 2.6	35.5 ± 2.9	36.3 ± 2.8	37.1 ± 2.7	8.35	< 0.001**	0.063

Note. Data are presented as Mean ± Standard Deviation (M ± SD); Group sizes are shown as N (%). One-way ANOVA was conducted to examine the effect; All comparisons were significant at p <0.001; η^2^ represents the partial eta-squared effect size.

### Predictors of subclinical ischemic heart disease risk

Table 6 indicates that OSA risk (SBQ), age, male gender, diabetes, hypertension, and a family history of heart disease were identified as significant positive predictors of the INTERHEART Risk Score (IHRS). In contrast, weekly physical activity was a protective factor. In particular, increased SBQ scores correlated with increased IHRS (B = 0.32, 0.17, and p < 0.001). Higher IHRS was also associated with older age (B = 0.41, 2 = 0.15, p = 0.002) and being male (B = 0.88, 2 = 0.14, p = 0.001). IHRS scores were further elevated by diabetes (B = 0.72, p = 0.004), hypertension (B = 0.65, p = 0.016), and family history of heart disease (B = 0.54, p = 0.031). Weekly physical activity decreased IHRS (B = -0.42, -0.10, p = 0.009), which provides a protective effect against the subclinical risk of ischemic heart disease.

**Table 6:** multiple linear regression predicting INTERHEART Risk Scores from OSA and clinical factors (N = 500)

Predictor	B	SE	β	t	p	95% CI LL	95% CI UL
Constant (INTERHEART Risk Score questionnaire)	30.25	1.20	-	25.21	< 0.001**	27.89	32.61
STOP-BANG questionnaire (SBQ)	0.32	0.09	0.17	3.76	< 0.001**	0.15	0.49
Age	0.41	0.13	0.15	3.15	0.002**	0.16	0.66
Gender (Male = 1, Female = 0)	0.88	0.26	0.14	3.38	0.001**	0.37	1.39
Diabetes	0.72	0.25	0.11	2.88	0.004**	0.23	1.21
Hypertension	0.65	0.27	0.10	2.41	0.016*	0.12	1.18
Family history of heart disease	0.54	0.25	0.09	2.16	0.031*	0.05	1.03
Weekly physical activity (protective)	-0.42	0.16	-0.10	-2.63	0.009**	-0.73	-0.11

Note. Multiple linear regression was conducted to identify predictors of INTERHEART Risk Score Questionnaire (IHRS) Scores; Values include unstandardized coefficients (B), 95% confidence intervals (CI), standard error (SE), standardized beta coefficients (β), and p-values; A p-value < 0.05 or <0.01 was considered statistically significant, N = 500.

### Predictive model of subclinical ischemic heart disease risk

**Figure 1:**
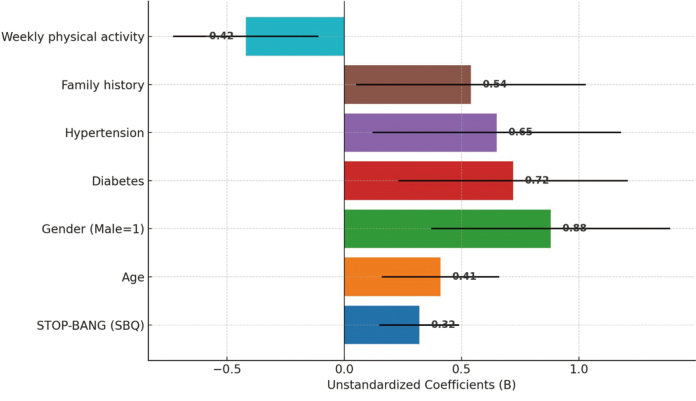
Regression coefficients and 95% confidence intervals for predictors of subclinical ischemic heart disease risk

The regression coefficients, along with the 95% confidence intervals for the predictors of the INTERHEART Risk Score (IHRS), are illustrated in Figure 1. The findings indicate that age, male gender, diabetes, high BP, family history of heart disease, and high scores in STOP-BANG (SBQ) were all positively related to the increased IHRS, which implies more cardiovascular risks. Contrastingly, the physical activity per week exhibited a negative association, indicating that it provided a protective effect. Interestingly, gender, diabetes, and hypertension significantly contributed positively, whereas physical activity was the only significant protective factor against increased IHRS scores.

### Association between family history and physical activity

Table 7 indicates that family history of heart disease has a significant relationship with levels of physical activity, χ2(3) = 16.8, p < 0.001. Participants who reported having a family history of heart disease were more likely to be sedentary (215, 65%) and less likely to engage in moderate-to- vigorous physical activity (53, 16%) than those without a family history, who were more likely to participate in moderate-to-vigorous activities (145, 87%). This suggests that physical activity levels are lower in individuals with a family history of heart disease.

**Table 7:** association between family history of heart disease and weekly physical activity (N = 500)

Family history of heart disease	Sedentary	Light (<150 min/week)	Moderate (≥150 min/week)	Vigorous	Total	χ^2^ (df = 3)	p
Yes	215 (65%)	65 (20%)	43 (13%)	10 (3%)	333 (100%)	-	-
No	4 (2%)	18 (11%)	65 (39%)	80 (48%)	167 (100%)	-	-
Total	145 (29%)	280 (56%)	61 (12%)	14 (3%)	500 (100%)	16.8	< 0.001**

Note. Data are presented as N (%); the Chi-square test was used to assess the relationship between family history of heart disease and physical activity frequency; Statistical significance was considered at p < 0.05

## Discussion

The research has investigated the association between the risk of subclinical ischemic heart disease (IHD) and obstructive sleep apnea (OSA) in obese adults. In our study, a moderate, positive correlation was found between OSA risk and subclinical ischemic heart disease risk (r = 0.36, p < 0.001), indicating that greater OSA risk is associated with higher cardiovascular risk. This result aligns with existing evidence that OSA is an independent risk factor for cardiovascular disease, emphasizing the need to screen and intervene early^15)^.

OSA risk was significantly higher in males, consistent with previous studies showing greater OSA severity among men, while women generally have a milder form^16)^. Similarly, male cardiovascular risk was slightly higher, aligning with evidence that traditional risk factors such as hypertension, diabetes, and smoking disproportionately affect men^17)^.

OSA and cardiovascular risk were both found to be age-related, with the highest risk observed between 50-59 years, which aligns with the findings that ageing is a cause of airway collapsibility, visceral fat deposition, and metabolic dysfunction^18, 19)^.

We found that OSA risk, older age, and male gender were risk factors that elevated cardiovascular risk, indicating the importance of these variables in increasing INTERHEART scores^15, 17, 19)^. We also found that diabetes was associated with a higher risk of CVD, which is correlated with the evidence that individuals with diabetes have a high risk of cardiovascular events. This implies the importance of controlling and supervising diabetes to reduce cardiovascular morbidity and mortality^20)^. We found out that hypertension was associated with cardiovascular risk, which can be supported by the evidence that high blood pressure is among the major contributors to CVD. This justifies the importance of managing blood pressure to reduce cardiovascular morbidity and mortality^21)^. Family history of heart disease was associated with a higher cardiovascular risk in our study, which was consistent with other studies demonstrating that parental CVD history positively but insignificantly predicts future CVD in children. This emphasises the importance of considering family history in the process of ascertaining cardiovascular risk^22)^. In our study, weekly exercise was found to reduce cardiovascular risk, which aligns with the evidence that exercise is a protective factor, particularly among older adults and individuals with metabolic comorbidities^23)^.

Those participants who had a family history of heart disease were also more likely to be sedentary, which is consistent with findings that high-risk persons tend not to adopt healthier lifestyles. This highlights the need for targeted interventions that promote physical activity among high-risk groups^24)^.

Some limitations to this study must be taken into consideration when interpreting the findings. The design is cross-sectional, which does not allow for determining causality between OSA and subclinical ischemia. Convenience sampling from selected hospitals may have introduced selection bias, limiting generalizability to the broader obese adult population. In addition, the use of English-only questionnaires may have excluded non-English speakers or affected comprehension, further limiting representativeness. Although validated, questionnaire-based measurements, such as the STOP-Bang and INTERHEART Risk Score, may not adequately reflect the true burden of disease compared with objective diagnostic tests, such as polysomnography or cardiovascular imaging. Moreover, potential confounding variables, such as dieting, socioeconomic status, and psychosocial stressors, were not fully measured and may have affected the observed correlations. Another limitation is the underrepresentation of younger adults, which restricts conclusions about OSA and cardiovascular risk in early adulthood. Lastly, the predominance of males in the study might have biased the gender comparison, and thus, conclusions could not be drawn on women.

### Recommendations for future research

These limitations can be overcome in future studies by employing longitudinal designs to elucidate causal mechanisms and utilizing objective diagnostic instruments to provide more valid measures of OSA and subclinical IHD. The generalizability of the findings could be improved by larger and more representative samples across multiple countries, and the introduction of other variables pertaining to lifestyle, psychosocial, and environmental determinants would allow for a more comprehensive picture of risk. Interventional studies on the effects of weight loss, structured exercise, and OSA-based treatment, such as Continuous Positive Airway Pressure (CPAP), on cardiovascular outcomes should also be conducted. Additionally, researching the genetic and cultural risk factors of South Asians may be beneficial for prevention and control measures.

## Conclusion

This research study provides evidence that there is a significant risk of subclinical ischemic heart disease that is moderately associated with the risk of OSA among the obese. Older age, gender (male), diabetes, high BP, and a family history of heart disease complicate this risk, which apparently can be mitigated by regular exercise. These findings highlight the importance of routine OSA screening and cardiovascular risk assessment in obese patients. Early identification allows for targeted interventions, such as weight management, lifestyle modification, and treatment of OSA, which may help prevent progression to overt cardiovascular disease.

## Author contributions

CI, EN, and AA conceived and designed the study. BK and MOP coordinated data collection across international sites and ensured data integrity. GI and QG contributed to data acquisition and data entry. OO and RAN performed statistical analyses and data interpretation. MAS assisted in manuscript drafting and reference organization. KH supervised the project, provided critical input during manuscript preparation, and served as the corresponding author. All authors read and approved the final version of the manuscript.

## Conflicts of interest statement

The authors declare that there are no conflicts of interest.
